# Factors explaining men’s intentions to support their partner’s participation in cervical cancer screening

**DOI:** 10.1186/s12905-022-02019-y

**Published:** 2022-11-11

**Authors:** Jyoshma Preema Dsouza, Stephan Van den Broucke, Sanjay Pattanshetty, William Dhoore

**Affiliations:** 1 Psychological Sciences Research Institute, Université catholique de Louvain, Ottignies-Louvain-la-Neuve - Louvain-la-Neuve, Belgium; 2 Prasanna School of Public Health, Manipal Academy of Higher education, Manipal, India; 3 Institute of Health and Society, School of Public Health, 1200 Woluwe-Saint-Lambert, Belgium

**Keywords:** Male involvement, Cervical cancer, Secondary prevention, Health behaviour, Beliefs

## Abstract

**Background:**

Cervical cancer represents a high burden of disease. Many women in low- and middle-income countries face opposition from their partners and families to undergo cervical cancer screening. Identifying the social, cultural, and psychological factors that underly the opposition to screening by male partners is an important step towards reducing barriers for men to support their wives’ participation in cervical screening. This study explored the role of structural and psychological factors deriving from theoretical models as determinants of Indian men’s opposition to their partners being screened for cervical cancer.

**Methods:**

A survey among 500 sexually active males was conducted between April 2020 and August 2020 to measure knowledge of cervical cancer and screening, awareness of screening possibilities, attitude towards screening, perceived barriers to screening, and health literacy. Regression analysis was performed to assess which of the potential factors contributed to the intention to support their wives’ screening.

**Results:**

The majority of participants had very poor knowledge and awareness about cervical cancer and screening procedures, tended towards a negative attitude towards screening, and perceived several structural barriers. Attitude towards the screening procedure and routine participation in general screening significantly predicted their intention to support their wives’ screening for cervical cancer. Education moderated the association between knowledge and awareness and the intention to support their wives’ screening.

**Conclusion:**

As women often rely on their spouses’ financial and emotional support of cervical screening, there is a need for men to be encouraged to support their wives’ screening participation. Programs to encourage men to support their wives’ cervical screening should focus on their attitude towards screening, educate about cervical cancer and screening procedures, and reduce perceived barriers.

## Introduction

Cervical cancer is the fourth most common cancer among women and represents one of the largest global burdens of disease [1]. The mortality due to cervical cancer remains very high worldwide, especially in Low- and Middle-Income countries (LMICs), due to the ageing and growth of the population and the variations in the occurrence and distribution of risk factors linked to socio-economic development [2, 3]. In the year 2020, there were 0.6 million new cases of cervical cancer in the world, most of them occurring in the Asian region [4], with the majority of the deaths taking place in LMICs [1]. India alone contributes to up to one-third of the burden of cervical cancer in Asia, with 0.9 million cases and 60, 000 deaths in 2020 [5].

Whereas most cancers are difficult to prevent as they are associated with multiple causal agents [6], cervical cancer can be prevented to some extent by the appropriate and consistent use of condoms which prevent the transmission of the Human Papilloma Virus (HPV) [7]. In addition, the discovery of a vaccine against cervical cancer in 2006 has increased the possibility to prevent the disease. Besides these two forms of primary prevention, the infection can also be detected at an early stage before it progresses to cancer. In its *Global strategy to accelerate the elimination of cervical cancer*, the World Health Organisation therefore proposes three main strategies to reduce the burden of cervical cancer, with targets to be reached by 2030: (i) Vaccination of 90% of girls against HPV by the age of 15; (ii) Screening of 70% of women with a high-performance testing method by the age of 35, and again by the age of 45; and (iii) Treatment of 90% of women with pre-cancer, and management of 90% of women with invasive cancer [8].

Vaccination against HPV has been initiated in most developed countries, but is more difficult to implement in LMICs due to the high vaccine cost [9]. Although vaccination is approved in India, only the affluent can choose to be vaccinated as each dose of the vaccine costs about 25–37 USD. Those living in poverty do not have the choice to get vaccinated as it is not yet included in the country’s immunization programs due to various reasons [10]. Factors like low social acceptance of the vaccine, lack of protection against certain strains of the virus, and uncertainty about the duration of the protection provided by the vaccine are additional barriers to large-scale vaccine implementation against cervical cancer in India [11]. As a consequence, screening remains the main prevention strategy against cervical cancer in the country.

Screening for cervical cancer using methods recommended by WHO has been tested and found feasible for implementation in India but has not yet been rolled out in the form of systematic screening. The National Program for Prevention and Control of Cancer, Diabetes, Cardio-vascular disease and Stroke (NPCDCS) includes screening women for cervical cancer at community health centres, has been initiated in a few districts and is expected to be expanded throughout the country. Yet despite the availability of opportunistic screening and the initiation of the NPCDCS, CCS uptake remains low [12, 13]. Apart from certain characteristics of the health system that pose structural barriers to the implementation of the program [14], this low participation in screening by women is likely due to psychological and social factors [15], including inadequate knowledge and awareness of the disease and of screening possibilities, or negative attitudes towards screening [12]. Besides these, the social environment including the family and community can also have an impact on the decision to be screened [15]. In this regard, stigma, community traditions, religious beliefs, and lack of support from a partner have been mentioned as important influencers in decision-making [16].

Men play a significant role in the transmission of the HPV that causes cervical cancer. However, in education about the disease little attention is given to the men’s role, despite the WHO’s recommendation to involve males in CCS education [17]. Several studies have also shown that women’s decision to get screened relies on the opinion of their husbands or partners [18, 19]. This is probably even more the case in LMICs like India [15, 20], where decision-making regarding a woman’s health mostly lies with her husband. Yet while the few studies that have investigated this issue show poor knowledge among men about cervical cancer in LMIC [21–24] in LMIC, not many studies have been conducted on these countries to assess the opinion and knowledge of males on cervical cancer prevention.

Considering the importance of the male partner’s role in the promotion of cervical cancer screening, it is important to identify the factors that determine the partners’ support for screening. Among the various factors that could be considered in this regard are knowledge about cervical cancer and screening, health literacy, attitude, perceived norms, perceived barriers, and habits.

Health literacy refers to a person’s knowledge, motivation, and competencies in accessing, understanding, appraising, and applying health-related information with a view to making decisions related to health [25]. Poor health literacy is associated with lower participation in screening programs, suboptimal use of preventive services, and lower engagement in health promoting behaviours [26]. There are also potential links between health literacy and cervical cancer screening [27].

Attitude, perceived norms, and perceived barriers are social cognitive concepts that are central to behaviour theories that have been specifically developed to explain peoples’ health-related behaviour or intentions, such as the Health belief model (HBM) [28], the Protection Motivation Theory (PMT) [29], or the Theory of Planned Behaviour (TPB) [30]. These models can predict behaviours like colon or breast cancer screening [31, 32]. A recent systematic review confirmed that the HBM is the most widely used model to inform interventions to promote CCS behaviour, whereas the TPB is the most effective to predict CCS intention [33]. The TPB identifies three constructs - attitudes, subjective norm, and perceived behavioural control - as the main determinants of (the intention to) performing a health behaviour. Attitudes are defined as evaluative statements about an object, for example, an individual’s evaluative statements about CCS, based on outcome beliefs. Subjective normative beliefs refer to a person’s belief that a behaviour is (or is not) acceptable to others (e.g., partner, parents, peers, society) [34]. While such beliefs are subjective, the perception regarding the acceptability of a behaviour by others is more important than their objective influence. Perceived behavioural control refers to a person’s perception of the ease or difficulty of performing the behaviour of interest [34]. Studies have shown that adding additional constructs such as habit (i.e., one’s usual care-seeking behaviour, such as routinely participating in general screening) and ‘affect’ adds to the prediction of the behaviour [35]. A recent study that we conducted among females in India confirmed that although TPB is a better predictor of the intention to be screened, additional constructs like ‘structural barriers’ and ‘habit’ could enhance the prediction of screening intentions [36]. A model that includes the two most predictive TPB constructs (‘attitude’ and ‘subjective norm’) in combination with ‘structural barriers’ and ‘habits’ provided the best prediction of Indian women’s intentions to be screened for cervical cancer.

Drawing on those findings, the present study intended to test whether this model (henceforth referred to as the modified TPB model) could also predict the intention of male partners to support their wives’ participation in CCS. In addition, we also wanted to assess if the effects of the model on the men’s intention to support screening are moderated by their knowledge and health literacy level. This, study specifically aimed to (a) explore the factors that influence male partners’ intention to support their wives’ participation in cervical cancer screening using the modified TPB; (b) assess the variance explained by each of the model’s components on the male partners’ intention to support their wives’ participation in screening; and (c) assess the moderating role of health literacy and education on the attitude and subjective norm towards screening.

## Materials and methods

### Study design and setting

A cross-sectional survey was conducted among male partners of sexually active women in Karnataka, a southern state of India. All methods were carried out in accordance with relevant guidelines and regulations. The state has a population of 61.09 million with nearly 50% of the females aged between 15 and 44 years. Nearly 62% of the population lives in rural areas. The literacy rate of males dwelling in rural areas is about 78% and of those dwelling in urban areas 90%. Cervical cancer contributes to 13% of cancers [37], but only 0.5% of women in the state have undergone cervical cancer screening [38] and the average cervical cancer examination rate is very low [39].

### Sample size and method

The study collected responses from a representative sample of sexually active males aged between 20 and 60 years. Individuals available at the time of data collection and able to read Kannada or English were included in the study. A sample size of 385 was calculated using Cochran’s formula considering a 50% response rate: Sample size = Z^2^ (p*q)/*d*^2^, with the estimated population proportion (p) of 50%, q=(1-p), the margin of error (d) as 5% and Z value 1.96  for 95%confidence level. The predicted variance of 50% was based on the population proportion (i.e., p = 0.5 yields an adequate sample to represent the population), since no similar studies existed for the given population.

As a first step, health facilities (public hospitals or tertiary screening centres) that conduct opportunistic screening for cervical cancer for less or no cost (hereafter referred to as screening centres) were identified in each district with the help of District health authorities, who provided a list of such centres. This was done to reduce the influence of barriers related to the availability of and affordability to screening. In a second step, two regions were identified, one accessible and one inaccessible to the screening centre, based on judgemental sampling to assess accessibility barriers. In the final step, individuals from both regions were approached using a consecutive approach.

Data collection was done by community health workers (ASHAs or Accredited Social Health Activists) who were trained in the data collection procedure and provided with a set of written instructions. The data collection took place under the supervision of the researcher who could be contacted whenever required.

### Participants

The study included 500 sexually active men who were willing to participate in the survey and familiar with the Kannada or English language. The participants from rural and urban communities were approached by the health workers using a consecutive sampling approach. The participant characteristics are presented in Table 2. The mean age of the participants was 41 years (SD 8.5). Most participants were employed (98.8%) and had at least completed secondary education (73%). Only a few (0.4%) had been trained in a health care profession. Although three out of four participants (76%) did not have a health insurance (76%), 53% of them claimed to undergo routine health check-ups (blood pressure, blood sugar, serum cholesterol, etc.) irrespective of symptoms, and 64.2% reported to have no difficulties to pay for these check-ups. The majority of participants (87%) declared that the decision related to health care expenditure for women in their families was taken by them (male partner) or by another family members (in-laws or parents).

### Conceptual model

**Fig. 1:**
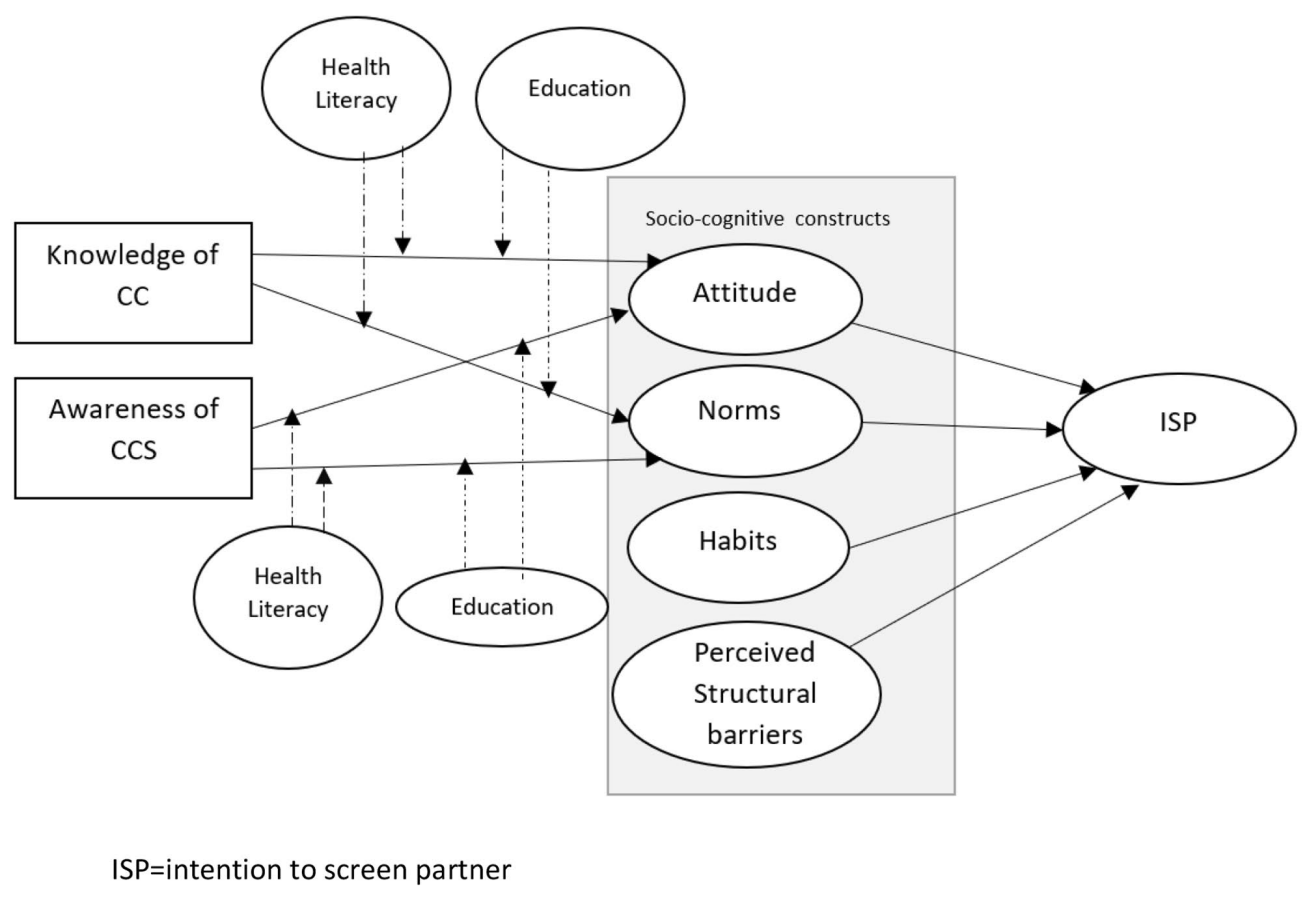
Conceptual model for male partners’ intention to support their wife’s participation in cervical cancer screening. (ISP = intention to screen partner)

The modified version of the TPB that was used for this study [33, 36] holds that the men’s intention to support their female partner’s cervical cancer screening (ISP) is influenced by attitude, subjective norms, and habitual screening behaviour, with attitudes and subjective norm in turn being influenced by knowledge, awareness of cervical cancer and routine screening participation (Fig. 1).

The construct of perceived behavioural control, which is normally part of the TPB, was not included in the model, since screening participation is a behaviour that is performed by the women, and as such not under the male partner’s control. Instead, perceived structural barriers to screening (e.g., financial cost, time, accessibility, or health system characteristics that could hinder the wives’ screening) were added to the model. Health literacy and education were added as moderating variables, as they significantly influence screening behaviour and knowledge.

### Questionnaire

For the data collection a structured questionnaire was developed, based on the adapted version of the TPB informed by a systematic review, a qualitative study, and a validation study conducted at earlier stages of the research project [15, 33, 40]. In addition to socio-demographic characteristics, knowledge about cervical cancer and screening, and CCS intention, it measured knowledge and awareness about cervical cancer and screening, attitude towards the wife’s participation, subjective norm, habits, health literacy, and structural barriers preventing the wife’s participation in cervical screening. The questionnaire items were in the form of close-ended questions. They were developed in English and translated into Kannada with the help of language experts, and pretested for cultural relevance, comprehension, and clarity, with adaptations made when necessary.

The resulting questionnaire consisted of five sections as shown in Table 1. The first section asked for the participants’ demographic and background characteristics. The second section measured knowledge about cervical cancer and screening through eight questions (5 on cancer, 3 on screening) with multiple answers from which the participant had to choose the right one, yielding a score between 0 and 16. The reliability (KR-20) for the knowledge scale was 0.88, which can be considered as good [41]. The third section measured the theoretical constructs of the modified TPB model, i.e., attitude towards CCS (7 items), subjective norms related to cervical cancer and screening (6 items) and perceived structural barriers to screening (5 items). Items for in the form of statements to be scored on a 5-point Likert scale ranging from ‘strongly agree’ to ‘strongly disagree’, with higher scores representing a higher level of the variable. Habit of participating in health screening was assessed by dichotomous scales (yes/no) asking participants if they engaged in regular check-ups of blood pressure, blood sugar, etc. even if they did not have any symptoms. Cronbach’s α for the attitude, subjective norms and structural barriers scales were 0.70, 0.72, and 0.83, respectively. The fourth section of the questionnaire measured health literacy using the HLS-IND-KAN-Q16 and HLS-IND-ENG-Q16 (25), which is a 16-item validated questionnaire for a Kannada and English-speaking population based on the HLS-EU questionnaire [42]. It contains questions about the ease or difficulty to perform various activities related to health care, disease prevention and health promotion, to be scored on a 5-point Likert scale (very difficult, difficult, don’t know, easy, and very easy). A standardized Health Literacy index score out of 50 for each participant is calculated using the formula HL = (Average − 1) x (50/3), with an index score greater than 33 considered as adequate [43]. Finally, the participant’s intention to support his wife’s participation in cervical cancer screening was measured on a dichotomous scale (yes/no), as a dependent variable for the analysis.

**Table 1:** Questionnaire items included in the survey

*Section*	*Category*		*Variables*	*Items*
1	Socio-demographic characteristics		Age, income, level of education, employment status, training in a health care profession, as well as access to health care, health insurance, ability to cover health care costs, routine health check-ups, and information regarding health care decisions for women in the family.	10
2	Knowledge related to the disease (cervical cancer)		Etiology of cervical cancer (HPV as a cause of cervical cancer), risk factors (history of cancer, unprotected sex, etc.), warning signs of cervical cancer (foul-smelling discharge, bloody discharge between menstrual cycles, pain or bleeding during intercourse, etc.) Having heard of cervical cancer Known someone with cervical cancer	5
	Knowledge about screening		Presence of a test to detect HPV infection Need to undergo screening test irrespective of symptoms Presence of any symptoms in the past and screening	3
3	Model-based items			
	Attitude about screening		e.g., ‘Cervical cancer screening causes pain’, ‘I don’t know how it is done’, ‘I am afraid of being diagnosed’	7
	Subjective Norms related to cervical screening		e.g., ‘The family objects’, ‘I don’t know anyone who did the test’, ‘I don’t think my religion allows me’	6
	Health Habits		Engagement in routine screening for hypertension, diabetes, cholesterol, etc(irrespective of symptoms)	1
	Structural barriers to screening uptake (relayed to cost, time, accessibility, health system characteristics)		e.g., 'screening test is expensive’, ‘test takes too much time’ ,‘ unable to travel to hospital for screening’, ‘rude health professionals’ etc	5
4	Health literacy measuring tool		HLS-IND-KAN-Q16	16
5	Intention to support wife to undergo screening		‘I intend to support my wife to undergo screening’	1
				Total = 54

### Data analysis

Data analysis was done in SPSS version 25.0. Frequencies with percentages and mean scores with standard deviations were provided as descriptive statistical measures. Bivariate analysis was performed using chi-square to test the association between categorical independent variables and intention to support the wife’s CCS as a dichotomous outcome, and independent t-tests to measure the differences on the continuous variables between men with and without the intention to support screening. Next, a series of logistic regression analyses were performed to assess the relative contribution of different factors to the male partners’ intention to support screening and the variance explained by the models, with the proportion of explained variance given by Nagelkerke’s pseudo R square. In a first regression model, knowledge and awareness were regressed on the intention to support the wife’s screening as a (dichotomous) dependent variable. In a second model, attitude, subjective norms, habit and perceived structural barriers were added as predictor variables, and both models were compared to assess the added variance. In a third step, the potential moderating effect of health literacy and education on the association between attitude and subjective norms on the one hand and intention on the other hand was tested using PROCESS macro in SPSS 25.0 bootstrapping analyses with 5000 bootstrapped samples to obtain reliable 95% confidence intervals.

## Results

### Knowledge and experience with cervical cancer

The majority of the men (68%) had a positive intention to support their partner’s participation in cervical cancer screening. Additionally, 12.8% of individuals claimed that their women had had one or more warning signs of cervical cancer, and 3.8% claimed their partners had suffered pain or itchiness in the vaginal region, foul smelly vaginal discharge (3.6%), post-menopausal bleeding (3.4%), bleeding between menstrual cycle (2.6%), or post-coital hemorrhage (1.4%). Men who completed secondary education had a significantly more positive intention to support their wife’s participation in screening than those who only had primary education. A small proportion of the participants (0.4%) who had been trained in a health care profession had a positive intention, but the difference was not significant compared to the untrained individuals (Table 2).

**Table 2:** Socio-demographic characteristics and facilitator scores of men with and without intention to support their partners’ participation in screening for cervical cancer

***SOCIO-DEMOGRAPHIC VARIABLES***	TOTAL (N = 500)	CCS Intention		***p-value***
		NO (n = 161)	YES (n = 339)	
**Age** (Mean, SD)	41.58 (8.5)	42.63 (8.5)	41.09 (8.5)	*NS*
**Income** (x 1000 INR) (Mean, SD)	16.68 (6.9)	16.05(6.70)	16.98(7.02)	*NS*
**Employment** (%)				
Unemployed	6 (1.2)	2 (1.2)	4 (1.2)	*NS*
Employed	494(98.8)	159 (98.8)	334 (98.8)	
**Education** (%)				
No secondary education	135 (27)	55 (34.2)	80 (23.6)	p < 0.05, df = 1, Χ²=6.17
Secondary education	365 (73)	106 (65.8)	259 (76.4)	
**Training in health care profession** (%)				
no	498 (99.6)	161 (100)	337 (99.4)	*NS*
yes	2 (0.4)	0 (0)	2 (0.6)	
**Health insurance** (%)				
no	377 (75.4)	126 (78.3)	251 (74)	*NS*
yes	123 (24.6)	35 (21.7)	88 (26)	
**Ease of health care expenditure** (%)				
difficult	321 (64.2)	65 (40.4)	114 (33.6)	*NS*
easy	179 (35.8)	96 (59.6)	225 (66.4)	
**Habit** (%)				
no	265 (53)	107 (66.5)	158 (46.6)	p < 0.001 df = 1, Χ²=17.27
yes	235 (47)	54 (33.5)	181 (53.4)	
**Healthcare-expenditure decision-making** (%)				
others	418 (83.6)	134 (83.2)	284 (83.8)	*NS*
woman herself	82 (16.4)	27 (16.8)	55 (16.2)	
***FACILITATOR VARIABLES***				
		NO(n = 358)	YES(n = 249)	
**Had symptoms**				
no	436(87.2)	141 (87.6)	295 (87)	*NS*
yes	64 (12.8)	20 (12.4)	44 (13)	
**Known someone with Cervical cancer**(%)				
no	438 (87.6)	139 (86.3)	299 (88.2)	*NS*
yes	62 (12.4)	22 (13.7)	40 (11.8)	
**Health literacy** (Mean, SD)	29.05 (5.5)	25.6 (5.4)	30.6 (4.8)	p < 0.05
limited	394 (78.8)	150 (93.2)	244 (72)	
adequate	106 (21.2)	11 (6.8)	95 (28)	

Knowledge about cervical cancer among men was poor. Most participants (66%) had never heard of cervical cancer, and only 12% had known someone with cervical cancer. Only 7.8% of the participants believed that cervical cancer is caused by a virus that is transmitted through sexual contact, and 21% were not aware of any of the risk factors of cervical cancer. More than half of the respondents (61%) did not know about the warning signs of cervical cancer, while about 40% identified chronic foul or blood-filled vaginal discharge as a sign of cervical cancer. The majority of respondents (95.4%) were unaware of the screening procedure, and 82% did not know that regular screening irrespective of warning signs was necessary.

The mean health literacy score of the participants in the study was 29.05, which is below the cut off score of 33 signifying a sufficient level of health literacy. Nearly 80% of the participants had limited health literacy. Those with a positive intention to support their wives’ participation in screening (ISP) had a significantly higher score for health literacy (30.6) than those without this intention (25.6), yet their average health literacy score remained in the ‘limited’ level.

With regard to the socio-cognitive factors measured in the theoretical model, most men had a negative attitude towards screening, with 8.2% of them even considering screening to be non-beneficial. Most participants (46%) were anxious about the procedure while others felt uncomfortable (30.4%) or were afraid of the outcome (35.8%). In terms of subjective norms, about 17% of the participants did not know anyone who had been screened for cervical cancer, 11% of them thought that it was socially unacceptable, and 8% thought that the families would not approve of screening. Norms were not significantly associated with intention. The most common perceived barriers to cervical cancer screening were health system-related (63%) followed by lack of time (27%), low accessibility to screening centers (20.6%), and cost (15.6%).

### Bivariate analysis

Analysis of the intercorrelations between the variables (Table 3) revealed that participants with better knowledge of cervical cancer were more aware of screening and more health literate. They also had a more positive attitude and scored higher for subjective norms (p < 0.05). Those who were more aware of the screening procedure were more knowledgeable and had higher scores on positive attitude and structural barriers, but lower on subjective norms (p < 0.05). Higher scores on subjective norms correlated positively with knowledge but negatively with positive attitudes and structural barriers, and a higher score on health literacy correlated positively with all variables except subjective norms.

Independent samples t-tests of the differences between participants with a positive or no intention to support their wife’s participation in screening revealed that the former were significantly more aware of the screening procedure, had a more positive attitude towards screening, perceived fewer structural barriers, and had significantly higher levels of health literacy. In contrast, the intention to support one’s wife’s participation in screening was not related to perceived norms.

**Table 3:** Bivariate analyses of the determinants of male partners’ intention to support their wives’ participation in cervical cancer screening

	ISP(Intention to screen partner)			Knowledge about cervical cancer	Awareness about CCS	Attitude	Subjective Norm	Structural Barrier	Health Literacy
	NO ISP Mean(SD)	ISP Mean(SD)	t-value (t-test)			Correlation coefficient (r)			
Knowledge about cervical cancer	2.76 (3.09)	3.65 (2.77)	-3.23						
Awareness about CCS	2.38 (0.68)	2.53 (0.97)	-1.81**	0.136**					
Attitude	25.96 (2.99)	28.65 (5.32)	-5.97**	0.142**	0.502**				
Subjective Norm	5.87 (1.37)	5.89 (1.34)	-1.64	0.364**	-0.169**	-0.184**			
Perceived structural barriers	12.27 (1.44)	11.19 (3.15)	4.14**	-0.035	0.462**	0.748**	-0.266**		
Health Literacy	25.66 (5.4)	30.65 (4.8)	-10.42**	0.168**	0.146**	0.301**	0.054	0.256**	

**p-value less than 0.005

### Multivariate analysis

Logistic regressions assessing the relative contribution of different factors to the male partners’ intention to support screening revealed, in the first step (Model 1, Table 4) that knowledge of cervical cancer and awareness of screening explained only 3.9% of the variance of the men’s intention (Adjusted R square = 0.035). The addition of attitude, subjective norms, habits, and perceived structural barriers (Model 2) increased the explained variance of the intention to 19% (adjusted R square = 0.179), with the socio-cognitive variables alone explaining up to 15% of the variance. Inspection of the odds ratios showed that a positive attitude (OR = 1.18) and especially the habit of regular health screening (OR = 3.04) are the main predictors of the man’s intention to support their wives’ participation in cervical screening, whereas knowledge of cervical cancer and awareness of screening procedures are only marginally significant, and subjective norms and perceived structural barriers do not contribute to the intention. It is noted, however, that the model does not have a good fit (Hosmer-Lemeshow χ²= 24.5, df = 8, p < 0.05), which is probably due to the high correlation between attitudes and perceived structural barriers (r = 0.748). When the latter variable is left out, as well as the subjective norm (which does not contribute significantly), the remaining model with awareness, attitudes and habits has a good fit (Hosmer-Lemeshow χ²=11.65, df = 8, p = 0.17) and explains 16% variance in the intention to support the partner’s participation in screening.

**Table 4:** Logistic regression analysis to predict male intention to screen partners for cervical cancer

	Model 1			Model 2		
	OR	95% CI	***p-value***	OR	95% CI	***p-value***
Knowledge about CC	1.11	1.03–1.19	0.003	1.08	0.99–1.17	0.062
Awareness about CCS	1.17	0.94–1.46	0.162	0.77	0.58–1.02	0.069
Attitude				1.18	1.09–1.27	< 0.001
Norm				1.01	0.85–1.19	0.888
Habit				3.04	1.98–4.65	< 0.001
Perceived structural barriers				0.98	0.86–1.11	0.792

Model 1: R²= 0.035 ,R²(adj) = 0.031, Hosmer-Lemeshow goodness-of-fit test: χ²= 34.7, d.f.= 7, p-value = < 0.001

Model 2: R²=0.189 ,R²(adj) = 0.179, Hosmer-Lemeshow goodness-of-fit test: χ²= 24.5, d.f.=8, p-value = 0.002

The moderation analyses using Hayes’ Process Macro to test if health literacy and education moderate the relationships between the variables impacting the men’s intention to support CCS revealed a significant moderator effect of education on the association between knowledge and awareness (dependent variables) and attitude (independent variable). The interaction between knowledge and the moderator (education) was statistically significant for attitude (β = 0.43, SE = 0.17, p = 0.0128, LLCI = 0.09; ULCI = 0.73). Similarly, the interaction between awareness and the moderator education was statistically significant for attitude (β= -1.77, SE = 0.47, p < 0.001, LLCI= -2.71; ULCI= -0.83). This suggests that a higher education level increases the effect of knowledge of the disease and of awareness of the screening procedure on having a positive attitude towards screening. Health literacy was not a significant moderator.

## Discussion

Screening is the main prevention strategy against cervical cancer in India, but despite the availability of opportunistic screening and the initiation of systematic screening within the National Program for Prevention and Control of Cancer, Diabetes, Cardio-vascular disease, and Stroke (NPCDCS), the uptake of screening remains low. One of the various factors that may influence screening uptake by women in the target group is the lack of support from their partners. While previous research has shown that women’s willingness to be screened for cervical cancer depends on their husbands’ opinion or approval [18, 44], a lack of such approval has been identified as a social barrier to participating in screening for many women in India [15, 19, 20, 45] and in other LMICs [46]. Therefore, the WHO recommends education of males to increase their willingness to encourage and support their partners [17]. Yet apart from knowledge and education, other factors may hamper men’s support for their wives’ participation in screening. As for women, the factors that influence the men’s decision-making regarding screening are manifold, and can best be understood by using conceptual models that acknowledge the role of both socio-economic factors such as age, income and education, and socio-cognitive factors like knowledge, attitudes, perceived norms, habits, or health literacy.

In accordance with these views, the present study aimed to identify the perceptions and beliefs of Indian men that could act as barriers to encouraging their partners to uptake cervical screening. To that effect, we relied on a modified version of the TPB that had been successful in predicting screening intention among Indian women in a previous study [34], and which included attitudes towards screening, perceived social norms, perceived barriers and regular screening practices for other health issues, while also considering knowledge of cervical cancer and screening and health literacy as potential moderators.

A first finding was that about 84% of the participants in the study claimed that decisions on healthcare for their wives were taken by themselves or other family members, confirming findings from previous research regarding male domination of health related decisions of women for financial [47] or emotional reasons [48]. Furthermore, participants who had completed secondary education were more likely to have a positive intention to support their wife’s screening, which is in line with other studies showing an association between education level and cancer screening in women [13].

### Knowledge and awareness of cervical cancer and cancer screening

With regard to socio-cognitive factors, our findings confirmed those of previous studies [49], including those that had been performed in Low-middle income countries [46, 50] that knowledge about the disease and awareness of the possibility to be screened are significant predictors of screening intention. This is similar to what has been found among females in this context [36]. In that regard, it is important to note that the overall knowledge about cervical cancer, its aetiology, risk factors, and early warning signs, was very low amongst the male participants in our study, which is consistent with the results of similar studies conducted earlier [15, 18, 22–24, 44, 51] and among women. We found that although most of the men who participated in the study had heard about cervical cancer, more than 90% did not know that cervical cancer is caused by the HPV virus transmitted through sexual contact, and that many were lacking in concrete knowledge about the disease and screening. Similar findings have been reported for other developing countries. For instance, in a study conducted in Uganda, it was reported that most men had heard of HPV but were unaware that it was transmitted through sexual intercourse [51]. While most men in this study identified having multiple sexual partners as a risk factor for cervical cancer, only 4.8% knew that condom use can reduce the risk for the disease. This could be due to the belief that the disease is a result of punishment for promiscuity and lack of fidelity towards one’s partner, which has been reported in other studies in India [16]. Thus, having heard of cervical cancer does not necessarily imply having an adequate knowledge about the disease. Not surprisingly, the National Family Health Survey of India shows that only 4.1% of the couples in the state of Karnataka use a condom as a contraceptive method [38], implying that there remains a great need to promote awareness about the ways to prevent HPV transmission among males. Moreover, a very low percentage of the men in our study (4.6%) were aware of the screening procedure, and more than half of them (56.5%) did not know that screening had to be done at regular intervals. This concurs with findings from other studies indicating that women who would benefit from cervical cancer screening are also often unaware of the need for regular screening and of the availability of opportunistic screening services [15]. This lack of awareness of screening procedures and the availability of screening facilities can be a significant barrier for males to support their wives to participate in screening [52–54].

### Psychological determinants of men’s willingness to support cervical cancer screening

Whereas knowledge and awareness remain important determinants of the willingness of men to support their wives’ screening for cervical cancer, they are not the only ones. Whereas the Theory of Planned Behaviour has been shown to be one of the better models to predict screening uptake for cervical cancer [33], previous research among Indian women suggested that intention to participate in cervical cancer can be improved by using a modified version of the model containing perceived structural barriers and habits (i.e., regular health checks) in addition to attitudes and subjective norms. Our findings revealed that these factors, combined with knowledge of cervical cancer and awareness of screening, explained up to 18% of the variance in the men’s intention to support their wives’ screening. Among these variables, attitude towards screening and the habit of having regular health checks were significant determinants of intention. These same factors were found to be the main socio-cognitive predictors of women’s intention to participate in screening [55, 56]. A reduced model with only attitude and habits along with awareness of screening procedures gave a better fit and explained a total of 16% variance in the men’s intention to support their wives’ cervical screening.

The significant role of attitude as a predictor of screening intention (in women) has been consistently found in other studies. In contrast, habit (defined here as regularly participating in routine health check-ups) has not often been included in studies on screening uptake, and a clear relationship between habit and behavioural intention has not been reported consistently for other health behaviours either [57, 58]. In this study, however, men who regularly underwent routine health check-ups themselves were twice as likely to have a positive intention to support their wives’ screening than those who did not undergo routine screening, suggesting that habit is a significant predictor of the men’s intention to support their wives’ screening. Subjective norm, on the other hand, was not found to be a significant predictor. This is comparable to the findings of a study of determinants of cervical screening uptake among Indian women [34], although other studies did show an effect of subjective norms on screening intention [56]. This difference in findings is not surprising given the variation in the conceptualization and operationalization of the constructs, the different context in which studies are carried out, and the nature and background of participants [33].

### The moderating role of health literacy and education

Besides the structural and socio-cognitive determinants of the male partners’ intention to support their wives’ screening, this study also investigated the moderating role of education and health literacy. Health literacy is an important quality that enables an individual to acquire, understand, evaluate, and apply health-related information, thus allowing them to make well-informed decisions regarding their health and that of their families. Similar to previous research [59], our study revealed a significant positive correlation between health literacy and knowledge of cervical cancer and awareness of screening procedures. Men with higher levels of health literacy tend to have a more positive attitude towards cervical cancer screening, which is similar to the direct relationship between health literacy and screening uptake among women [59, 60]. Moreover, the low level of health literacy that was found among the men who participated in this study is comparable to that of other studies among Indian males [61] and among women in a similar context. However, we did not find a confirmation of the hypothesized moderating role of health literacy on the relationships between knowledge and awareness on the one hand, and attitudes, subjective norms, habit, and perceived barriers on the other hand. In contrast, a significant moderator effect was found of education on the association between knowledge and awareness (dependent variables) and attitude (independent variable), suggesting that a higher education level increases the effect of knowledge of the disease and of awareness of the screening procedure on having a positive attitude towards screening.

### Structural barriers to screening uptake

Various structural barriers can prevent the utilization of a screening offer. Structural barriers that prevent CCS utilization in LMICs have been well recorded [62, 63]. These barriers also exist in India, as has been reported elsewhere [64]. In our study, health system-related barriers were identified by most men, also those who had a positive intention to support their wife’s screening. This means that reducing structural barriers can be helpful. Among the barriers that are mentioned are the fact that most men do not like the approach of the health professionals or think that the process is time-consuming. These findings coincide with those of Binka et al., [52] and Basu et al. [65] who mentioned indifference by health professionals and poor quality of care received in public hospitals. In India, cervical cancer screening is available for minimum cost in district hospitals and selected health centres, which sometimes charge a fee for the procedure. This explains the role of geographical and financial inaccessibility of cervical screening for many women, as highlighted in previous studies in the same context [15, 46, 63, 66–68]. But in addition to these physical and structural barriers, it is also important to address social barriers, for which the engagement of family and community in CCS prevention is key [12]. Men can be supportive and help encourage women to disclose issues related to early warning signs of cervical cancer and support her to be screened, as most women fear to discuss these issues [15]. A study conducted in Kenya and sub-Saharan Africa showed that men were willing to support their partner to uptake screening and wanted to know more about screening and cervical cancer prevention [21, 24]. Beneficiaries of the program in India and their partners are mostly unaware of the existing services [54]. Hence, awareness on cervical screening must target men and families and must include information on how to support women in this regard, as seen in a study conducted in Ghana [44].

### Study Limitations

This study is not without limitations. Firstly, as it concerns a questionnaire study it is likely that the data include a certain amount of bias. It is possible that the participants’ responses were biased by social desirability tendencies. Moreover, random bias may have been induced due to the fact that data were collected by health workers. Secondly, the study explored factors that influence men’s intentions to support their wives’ screening uptake, rather than their actual behaviour. Since intentions do not always translate into actual behaviour, it is not possible to infer to what extent the intentions would be put into practice. For that, it would be useful to measure the outcomes of the existing actions within the NPCDCS program via a cohort study, looking at actual behaviour. Finally, it should be noted that interactions exist between the different variables included in this study. While these interactions were accounted for in the statistical analyses that were performed, it would be interesting to also study their direct and indirect effects on the outcomes.

## Conclusion

To improve the uptake of screening for cervical cancer most interventions aim to inform, encourage, and convince women to participate in screening programs, but engaging women alone might not be sufficient. To overcome social barriers to screening, the role of the male partners and families also needs to be acknowledged, especially in cultures where health-related decisions are mostly taken by men or heads of the family. While it is likely that the same kinds of social, cognitive, emotional, and cultural factors that influence women’s decisions to uptake screening can influence men to support their partners’ screening, research about these factors is scarce. This study is one of the first to examine socio-cognitive and structural determinants of males’ support of cervical cancer screening, using a behavioural model as a conceptual basis. The results of this study suggest that in addition to effective and active education to inform both women and men on cervical cancer, screening recommendation, the benefits of screening, and the services that are available, it is also important to promote a positive attitude towards screening among men. Interventions that are set up to that effect should take the overall low health literacy of men and families into account. Therefore, it would be wise to implement screening at community level and to strengthen the capacities of community health centres to provide their service.

## Data Availability

The datasets generated and/or analysed during the current study are not publicly available due to ethical reasons but are available from the corresponding author on reasonable request.
